# Real-world assessment of Multi-Frequency Bioelectrical Impedance Analysis (MFBIA) for measuring body composition in healthy physically active populations

**DOI:** 10.1038/s41430-025-01664-4

**Published:** 2025-09-16

**Authors:** Adam W. Potter, Leigh C. Ward, Christopher L. Chapman, William J. Tharion, David P. Looney, Karl E. Friedl

**Affiliations:** 1 United States Army Research Institute of Environmental Medicine, Natick, MA USA; 2 School of Chemistry and Molecular Biosciences, the University of Queensland, St. Lucia, Brisbane Australia; 3 Department of Health Sciences, Faculty of Medicine, Health and Human Sciences, Macquarie University, Sydney, Australia; 4 Oak Ridge Institute for Science and Education (ORISE), Oak Ridge, TN USA; 5 Maximize Human Performance LLC, Framingham, MA USA; 6 CoachMePlus, Buffalo, NY USA

**Keywords:** Weight management, Translational research

## Abstract

**Background:**

Multi-frequency bioelectrical impedance analysis (MFBIA) methods offer reliable and moderately accurate estimates of body composition in tightly controlled conditions (prandial and hydration status, recent exercise, time of day).

**Objective:**

This study examined MFBIA reliability and validity in a real-world environment where these factors were not controlled.

**Methods:**

Regional and total body composition estimates by MFBIA (InBody 770) were compared to dual-energy X-ray absorptiometry (DXA) in 1000 healthy adults (667 men; 333 women), including fat mass (FM), percent body fat (%BF), fat-free mass (FFM), and visceral adipose tissue (VAT). In subsets, reliability was determined from duplicate MFBIA and DXA obtained within 1 week, and total body water (TBW) was compared to single-frequency BIA (SFBIA).

**Results:**

MFBIA demonstrated modest population-level agreement with DXA for total body FM (men, *r* = 0.93, bias −3.7 ± 2.6 kg; women, *r* = 0.96, bias, −1.9 ± 1.8 kg), %BF (men, *r* = 0.89, bias, −4.2 ± 3.0%; women, *r* = 0.92, bias, −2.8 ± 2.6%), and FFM (men, *r* = 0.95, bias, 3.4 ± 2.8 kg; women, *r* = 0.94, bias, 2.0 ± 2.2 kg). Regional correlations were highest for trunk FM (men, *r* = 0.92, CCC = 0.86; women *r* = 0.93, CCC = 0.93) and lowest for VAT (men, *r* = 0.74, CCC = 0.68; women, *r* = 0.74, CCC = 0.34). DXA and MFBIA regional and total assessments were highly reliable (DXA, ICC 0.990–0.998) and (MFBIA, ICC 0.987–0.995). TBW by MFBIA and SFBIA showed moderate agreement (men, *r* = 0.73, bias, −1.89 ± 3.31; women, *r* = 0.82, bias, −1.74 ± 2.01).

**Conclusion:**

This MFBIA system was shown to have high retest reliability and, when compared to laboratory methods, provides a moderately accurate method for measuring TBW and body composition (except for VAT) in uncontrolled conditions.

## Introduction

Bioelectrical impedance analysis (BIA) has advanced considerably since its first applications to body composition analyses [1, 2]. The underlying principle of BIA is that impedance (measured in Ω), comprised of the two components resistance (*R*) and reactance (*Xc*) (both in Ω), can be used to assess the water content of biological tissues [1, 2]. Whole body resistance measured at 50 kHz (R50), typically between the hand and foot, expressed relative to height as the resistive index (RI, height^2^/R50), is a good predictor of total body water content (TBW) [3, 4]. From this, other components of body composition, namely fat mass and fat-free mass (FM, FFM), can be estimated based on the assumption that total body water comprises 73% of the fat-free mass [5–7]. While useful at the population level, the assumption of normal hydration at the individual level is not always valid and other factors, such as posture, skin temperature, exercise-induced blood flow may change the electrical properties of tissue [8].

Variations in hydration status, such as dehydration or overhydration, can significantly affect BIA measurements due to changes in the body’s electrolyte concentration and fluid distribution [9]. Recent exercise can also influence BIA by altering fluid shifts and skin temperature [10]. Similarly, food consumption and gastric emptying can impact fluid balance and impedance values [11].

Multi-frequency BIA (MFBIA) represents an advance in BIA-based body composition analysis compared to single-frequency BIA (SFBIA). Both SFBIA (typically 50 kHz) and MFBIA utilize alternating current (AC) frequencies that allow current to pass through both extracellular and partially intracellular spaces [12]. However, the extent to which current flows through the intracellular space is frequency dependent. In contrast to SFBIA, the use of a larger range of frequencies in MFBIA (typically from 5 kHz to 1 MHz or higher) allows for the collection of a more detailed impedance profile across different tissue compartments and enables better differentiation or calculations based on segments and fluid compartments [13, 14]. Using measurements at multiple frequencies (e.g., 5, 50, and 250 kHz) provides added information on water compartments since at low frequencies current flows predominantly through the extracellular water space (ECW); while at high frequencies current can cross the cell membrane and flow through both ECW and the intracellular water (ICW), i.e., TBW [15]. Because a range of frequencies can sample different water compartments across various tissues, MFBIA has the potential to provide more accurate body composition estimates compared to SFBIA, particularly in populations with varying hydration levels. With the development of octopolar BIA devices, segmental analyses can be easily performed providing regional body composition estimates for arms, legs, and trunk separately and recombined for total body assessments [16, 17].

Given the expanding role of BIA in weight management programs, performance monitoring, and military readiness assessments, it is important to establish whether current MFBIA systems provide sufficient accuracy for large-scale applications specifically in real-world conditions. The US Army evaluated BIA technology for body fat standard enforcement in 1984 in the largest military body composition study ever conducted and determined that SFBIA available at the time added technological complexity and no advantage over circumference-based body fat estimation [6, 18]. Recently, Potter et al. reevaluated the suitability of modern MFBIA technologies to replace military height-weight tables and circumference-based predictions of body fat; they concluded that new methods and algorithms may have overcome previous drawbacks with variability in electrode placement, standardization of body position, and influence of biological variables affecting individual measurements [19, 20]. This also prompted a fresh look at the body composition metrics that are most relevant to health outcomes, sports performance, and military readiness, such as trunk or visceral adipose tissue (VAT) and FFM or muscle mass [21, 22].

The present study evaluated the reliability and accuracy of a widely used MFBIA system (InBody 770, InBody Co. Ltd., Seoul, Korea) in uncontrolled free-living conditions, offering critical insights into the feasibility and practicality of MFBIA technology for routine body composition evaluations beyond controlled laboratory conditions to replace conventional metrics such as body mass index (BMI) or other anthropometric methods such as waist circumference assessments. The primary hypothesis was that MFBIA in uncontrolled field conditions would provide similar reliability and accuracy to that observed in a recent laboratory study with tightly controlled conditions [23]. Additionally, the accuracy of MFBIA regional assessments, including arm, leg, trunk, and VAT, was assessed by comparison to a criterion method, dual-energy X-ray absorptiometry (DXA) measurements. In a subset sample, reliability was assessed by repeated measurements within a week, to estimate biological variability. In another subsample, TBW estimates from the MFBIA system were compared to those calculated from resistance at 50 kHz using a conventional tetrapolar arrangement.

## Methods

### Participants

Study participants included a total of 1000 healthy active duty US Marines (*n* = 667 men, *n* = 333 women). Individuals were recruited from the US National Capital Region (Virginia, Maryland, and Washington, DC), Camp Pendleton, California, and from Camp Butler, Okinawa, Japan. Prior to study-related activities, all participants provided written informed consent, and women were provided a rapid pregnancy test to establish the absence of detectable pregnancy. Study approval was granted by the US Army Medical Research and Development Command (Fort Detrick, Maryland) and US Marine Corps (Quantico, Virginia) Institutional Review Boards, protocol M10873, approved March 2021.

### Study design

All participants were assessed for body composition during a single-day visit (<1 h). For retest reliability of measures, a subset of participants (*n* = 117; 100 men and 17 women) were assessed for all the same measures during a second visit separated by 5–7 days. Participants were recruited with the instructions to attend the testing session “*whenever they were available*” within a normal weekday. Additionally, during screening individuals were told of the study intent to capture body composition under typical daily conditions. No specific instructions were provided regarding fasting, hydration status, physical activity prior to testing, or avoidance of diuretics such as caffeine or alcohol. Each individual wore athletic clothing and was asked to remove all jewelry and/or foreign objects. Measurements were taken of standing height, to the nearest 0.1 cm, using a calibrated stadiometer (Seca, Chino, CA). Weight measurements, provided by the MFBIA device, were checked for recording errors by comparison to weight obtained between systems during the same test session. Whole body composition measures were assessed by DXA and algorithms (iDXA, enCORE software (version 13.5) GE Healthcare, Madison, WI), and by a standing MFBIA (1, 5, 50, 250, 500 and 1000 kHz) (InBody 770, InBody Co. Ltd., Seoul, Korea). It is important to note that the InBody 770 utilizes proprietary algorithms to estimate body composition parameters, and the specific equations are not disclosed by the manufacturer. Additionally, this approach did not involve impedance spectroscopy. Additionally, a subset of the sample (*n* = 685; 416 men, 269 women) was assessed for total body water (TBW, L) by a single-frequency (50 kHz) bioelectrical impedance analyzer SFBIA (Quantum IV, RJL Systems Inc., Clinton Township, MI) as previously described [20].

Outputs from the MFBIA were compared to the output DXA measurements for whole body values (FM, relative body fat (%BF), and FFM) as well as for regional FM and FFM measurements for the arms (left and right arms combined), legs (left and right legs combined), and trunk. Comparisons of the MFBIA output for TBW were compared to those calculated from the SFBIA system as well as for the main measures of *R, Xc*, and phase angle (*PhA*) at 50 kHz. We note that this is not a perfect comparison as SFBIA was measured with electrodes attached at wrist and ankle, while MFBIA was measured between palms and soles, altering the inter-electrode distance ~10 cm between the two systems. SFBIA TBW was calculated using sex-specific equations [24]. TBW was compared between the two systems as the primary component of body composition derived from resistance (versus secondary estimates that are derived from assumptions about the normal distribution of water in the FFM, etc.).

### Statistical analyses

Data were analyzed using R (Version 4.4.1; R Foundation for Statistical Computing; Vienna, Austria) [25] and reported as mean ± standard deviation (SD) unless specified otherwise. Agreement between MFBIA and DXA as well as between MFBIA and SFBIA for TBW was evaluated based on the bias (mean difference), SD of differences, Lin’s concordance correlation coefficient (CCC), mean absolute percentage error (MAPE), and root mean squared error (RMSE). Bland-Altman analyses were used to show bias and limits of agreement (LoA) within 95% of the measures [26]. Passing and Bablok Regression (PBR) [27] was used to assess agreement between methods. Using PBR, proportional differences are described by the slope (*B*_1_) and systematic differences by the intercept (*B*_0_); where *B*_1_ = 1 and *B*_0_ = 0 suggest perfect agreement. Intraclass correlation coefficient (ICC) partitioning out variance components using a linear mixed-effects model with random effects within participants was used to assess device reliability [28, 29].

## Results

A sample of 1000 healthy active duty US Marines (667 men, 333 women) was enrolled in the study. From the main sample, a subset of 117 (100 men, 17 women) provided repeated visits and these data were used for retest reliability comparisons. Participant characteristics (mean ± SD) were: men, *n* = 667, age 28.4 ± 7.4 years, height 176.6 ± 7.3 cm, body mass 86.3 ± 11.5 kg, BMI 27.7 ± 3.2 kg/m^2^ and women, *n* = 333, age 27.3 ± 6.8 years, height 162.8 ± 7.2 cm, body mass 67.9 ± 9.5 kg, BMI 25.6 ± 3.0 kg/m^2^. Body composition measures by DXA were 23.1 ± 6.3%BF, VAT 60.1 ± 45.2 cm^2^ and 31.7 ± 6.2%BF, VAT 37.1 ± 30.9 cm^2^ for men and women, respectively. The sample included self-reported race/Hispanic origin categories: Hispanic (29.5%), non-Hispanic white (58.0%), non-Hispanic black (11.1%), non-Hispanic Asian (1.1%), and non-Hispanic “other or multi-racial” (0.3%). More detailed descriptive statistics are shown in Table 1.

**Table 1:** Participant descriptive statistics.

Value	Description	Unit	Men	Women	All
Sample	*n*		667	333	1000
Descriptives	Age	years	28.37 ± 7.40	27.73 ± 6.76	28.17 ± 7.22
	Height	cm	176.57 ± 7.31	162.83 ± 7.23	172.27 ± 9.68
	Body mass	kg	86.25 ± 11.54	67.91 ± 9.51	80.50 ± 13.86
	Body mass index	kg/m^2^	27.66 ± 3.24	25.60 ± 3.00	27.01 ± 3.31
Race	Asian, non-Hispanic	# (%)	10	1	11 (1.1%)
	Black, non-Hispanic	# (%)	74	37	111 (11.1%)
	Hispanic	# (%)	183	112	295 (29.5%)
	White, non-Hispanic	# (%)	397	183	580 (58%)
	Other	# (%)	3	0	3 (0.3%)
Fat^a^	Arm fat	kg	2.16 ± 0.73	2.41 ± 0.72	2.24 ± 0.73
	Leg fat	kg	6.47 ± 2.22	8.37 ± 2.38	7.07 ± 2.44
	Trunk fat	kg	10.80 ± 4.58	10.19 ± 3.76	10.60 ± 4.35
	Total fat	kg	20.40 ± 7.22	21.83 ± 6.49	20.84 ± 7.03
	Relative body fat	%BF	23.12 ± 6.31	31.70 ± 6.18	25.81 ± 7.43
	Visceral adipose tissue	cm^2^	60.12 ± 45.27	37.12 ± 30.87	53.76 ± 43.02
Fat-free^a^	Arm fat-free mass	kg	9.47 ± 1.53	5.25 ± 0.94	8.15 ± 2.39
	Leg fat-free mass	kg	23.20 ± 3.19	15.91 ± 2.27	20.91 ± 4.48
	Trunk fat-free mass	kg	29.63 ± 3.66	21.26 ± 2.61	27.01 ± 5.14
	Total fat-free mass	kg	66.46 ± 8.06	46.10 ± 5.56	60.08 ± 11.98
Water^b^	Total body water	L	48.85 ± 6.08	33.75 ± 4.28	42.94 ± 9.16

^a^indicates measures obtained by dual-energy X-ray absorptiometry (DXA); ^b^indicates measures obtained from single-frequency bioelectrical impedance analysis (SFBIA) and equations by Sun et al. [17].

**Fig. 1:**
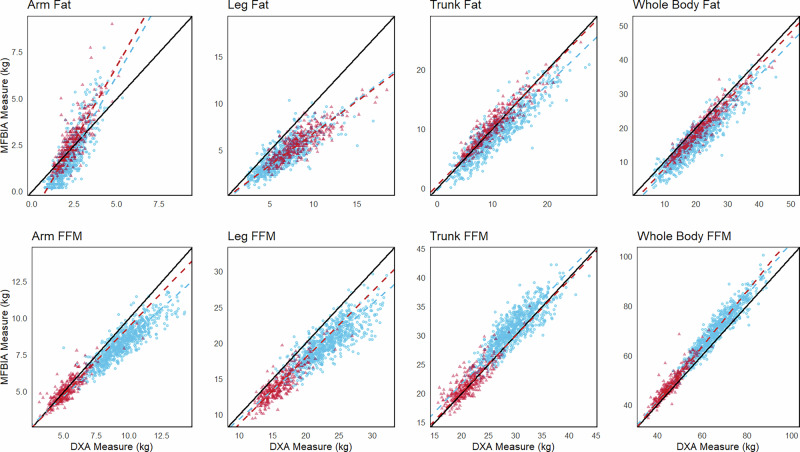
Passing and Bablok regression comparisons for fat and fat-free mass (FM, FFM) parameters from multi-frequency bioelectrical impedance analysis (MFBIA) measurements to dual-energy X-ray absorptiometry (DXA). Note: men are blue (circles); women are red (triangles), and regression lines are dashed.

**Fig. 2:**
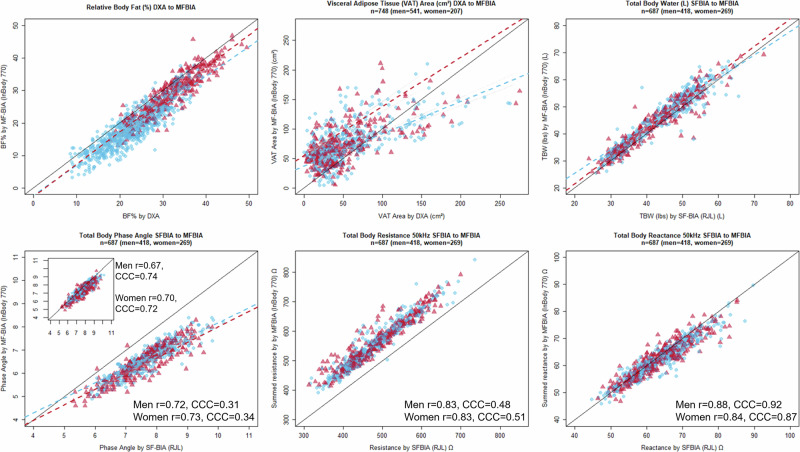
Comparison of selected estimations and measurements obtained from multi-frequency bioelectrical impedance analysis (MFBIA). Upper panel: MFBIA relative body fat (%BF) and visceral adipose tissue (VAT) measurements compared to dual-energy X-ray absorptiometry (DXA) measurements; MFBIA total body water (TBW) compared to single frequency bioelectrical impedance analysis (SFBIA) calculated TBW. Lower panel: measurements of phase angle, resistance, and reactance from MFBIA (calculated from the summed segmental data) compared to SFBIA. Inset graph represents calculated whole body phase angle based on segmental data for MFBIA; men and women are represented by blue circles and red triangles, respectively.

The MFBIA and DXA whole body and regional data for men and women were compared for precision and accuracy (Table 2; Figs. 1 and 2). Measures for total FM and FFM had comparable and generally high correlations (total FFM, men *r* = 0.95, CCC = 0.87 vs. total FM, *r* = 0.93, CCC = 0.82; women total FFM, *r* = 0.94, CCC = 0.94 vs. total FM, *r* = 0.96, CCC = 0.92). Additionally, the total body correlations for both FM and FFM were generally higher than each of their region values individually. Total body water comparisons between SFBIA and MFBIA for the sample were moderately correlated (men, *r* = 0.73, CCC = 0.82; women, r = 0.82, CCC = 0.84) (Table 2; Fig. 2). Of the comparisons, VAT had the lowest correlation (men, *r* = 0.74, CCC = 0.68; women, *r* = 0.74, CCC = 0.34). Additionally, while there was a moderate correlation in %BF (men, *r* = 0.89, CCC = 0.73; women, *r* = 0.92, CCC = 0.84), there was a relatively large negative bias (men, −4.2 ± 3.0; women, −2.8 ± 2.6) confirming a previously observed systematic offset [19, 23].

**Table 2:** Men and women assessment for accuracy and precision of multi-frequency bioelectrical impedance analysis (MFBIA) measurements to dual-energy X-ray absorptiometry (DXA).

	Variable	Sex	Bias ± SD	Bias *P*-value	LoA	*r*	CCC	MAPE	RMSE	PBR *B*_0_ (*p*-value)	PBR *B*_1_ (p**)
FM	Arms (kg)	Men	−0.43 ± 0.73	<0.001	−1.88–1.01	0.84	0.67	36.78	0.86	−1.75 (<0.001)	1.60
		Women	0.22 ± 0.72	0.002	−1.22–1.67	0.83	0.71	20.94	0.77	−1.27 (<0.001)	1.61
	Legs (kg)	Men	−2.08 ± 1.33	<0.001	−4.69–0.52	0.65	0.49	32.14	2.47	−0.02 (0.004)	0.71
		Women	−2.63 ± 1.32	<0.001	−5.21 to −0.04	0.72	0.43	30.81	2.94	−0.02 (0.895)	0.68
	Trunk (kg)	Men	−1.51 ± 1.82	<0.001	−5.08–2.07	0.92	0.86	18.42	2.36	−0.30 (0.020)	0.89
		Women	0.20 ± 1.36	0.007	−2.46–2.85	0.93	0.93	11.25	1.37	0.52 (<0.001)	0.96
	Total (kg)	Men	−3.72 ± 2.63	<0.001	−8.88–1.44	0.93	0.82	20.23	4.56	−2.77 (<0.001)	0.96
		Women	−1.92 ± 1.83	<0.001	−5.51–1.67	0.96	0.92	10.37	2.65	−1.96 (<0.001)	1.00
	Relative (%)	Men	−4.22 ± 2.95	<0.001	−10.01–1.57	0.89	0.73	19.98	5.15	−4.46 (<0.001)	1.01
		Women	−2.83 ± 2.61	<0.001	−7.95–2.29	0.92	0.84	10.40	3.85	−5.47 (<0.001)	1.08
	Visceral (cm^2^)	Men	9.84 ± 30.70	<0.001	−50.33–70.01	0.74	0.68	70.80	32.21	19.80 (<0.001)	0.85
		Women	48.69 ± 23.93	<0.001	1.79–95.60	0.74	0.34	567.23	54.23	39.17 (<0.001)	1.28
FFM	Arms (kg)	Men	−1.19 ± 0.75	<0.001	−2.66–0.27	0.88	0.62	12.56	1.41	0.71 (<0.001)	0.80
		Women	−0.15 ± 0.48	<0.001	−1.09–0.79	0.86	0.85	7.34	0.50	0.18 (0.234)	0.93
	Legs (kg)	Men	−3.18 ± 1.84	<0.001	−6.78–0.43	0.82	0.50	13.59	3.67	1.34 (0.007)	0.81
		Women	−1.90 ± 1.19	<0.001	−4.23–0.43	0.86	0.62	12.14	2.24	−0.65 (0.114)	0.93
	Trunk (kg)	Men	1.32 ± 1.89	<0.001	−2.38–5.01	0.86	0.81	6.47	2.30	2.14 (0.002)	0.97
		Women	0.35 ± 1.50	<0.001	−2.58–3.29	0.84	0.83	5.59	1.54	0.82 (<0.001)	0.97
	Total (kg)	Men	3.38 ± 2.79	<0.001	−2.09–8.86	0.95	0.87	5.55	4.39	−1.20 (0.235)	1.07
		Women	2.00 ± 2.17	<0.001	−2.25–6.25	0.94	0.88	4.79	2.95	−3.41 (<0.001)	1.11
Water	Total (L)	Men	−1.89 ± 3.31	<0.001	−4.58–8.37	0.73	0.82	5.93	3.81	−0.07 (0.958)	1.05
		Women	−1.74 ± 2.01	<0.001	−2.21–5.69	0.82	0.84	5.80	2.66	−2.50 (0.056)	1.13

Bias is calculated as MFBIA–DXA. Total body water (TBW) is a comparison between MFBIA and single-frequency bioelectrical impedance analysis (SFBIA) computed from 50 kHz resistance measurements.

*FM* fat mass, *FFM* fat-free mass, Bias is calculated from SD, standard deviation of differences, *LoA* limits of agreement (within 95%), *r* Pearson correlation coefficient, *CCC* concordance correlation coefficient, *MAPE* mean absolute percentage error, *RMSE* root mean square error. MFBIA (InBody 770); DXA (iDXA, GE Healthcare); SFBIA (Quantum IV, RJL). *B*_0_ Passing-Bablok intercept; *B*_1_ Passing-Bablok slope, p**all *p*-values < 0.001.

Table 2 outlines sex grouped comparisons for body regions between the MFBIA and DXA, as well as between the MFBIA and SFBIA for TBW. Women had higher correlations than men for total FM (women, *r* = 0.96, CCC = 0.92 vs. men *r* = 0.93, CCC = 0.82 kg) and very close values for total FFM (women, *r* = 0.94, CCC = 0.88 vs. men *r* = 0.95, CCC = 0.87 kg). Both women and men had high correlations for trunk FM (men, *r* = 0.92, CCC = 0.86; women, *r* = 0.93, CCC = 0.93 kg) and moderate correlations for FFM (men, *r* = 0.86, CCC = 0.81; women, *r* = 0.84, CCC = 0.83 kg). Measures for VAT had the highest relative bias, the lowest correlation, and highest errors for both men (9.84 ± 30.70, *r* = 0.74, CCC = 0.68, MAPE = 70.8, RMSE = 32.2 cm^2^) and women (48.69 ± 23.93, *r* = 0.74, CCC = 0.34, MAPE = 567.23, RMSE = 54.23 cm^2^). Bland–Altman analyses show negatively skewed LoA for %BF (men −10.01 to 1.57, women −7.95 to 2.29%), and wide LoA for VAT (men −50.33 to 70.01, women 1.79–95.60 cm^2^) (Table 2).

Generally, the two system raw data outputs are not comparable, as one is taken standing while the other is supine and the measurement locations are not exactly the same. However, along with TBW, Fig. 2 shows comparisons between the SFBIA to the summed segmental data from the MFBIA 50 kHz measures for *PhA*, *R* and *Xc*. The MFBIA system reports segmental *PhA* values as well as a whole body 50 kHz *PhA* which are markedly different when compared to SFBIA. Phase angle technically represents the angular difference between the voltage and current, calculated as a ratio of *Xc/R*, therefore it can be calculated. For this plot in Fig. 2, shown is the comparison of *PhA* from the SFBIA to the reported whole body *PhA* of the MFBIA; while the inset plot shows the average 50 kHz segmental *PhA* values for the five segments compared to the SFBIA value. Figure 2 also shows a comparison of the SFBIA 50 kHz *R* and *Xc* to the summed segmental MFBIA values divided by two for arms and legs plus the trunk value (i.e., $\frac{{\rm{right\; arm}}+{\rm{left\; arm}}+{\rm{right\; leg}}+{\rm{left\; leg}}}{2}+{\rm{trunk}}$).

**Fig. 3:**
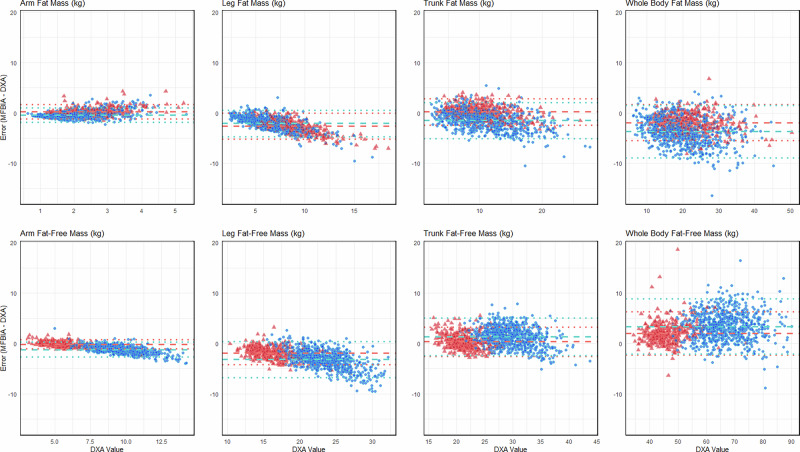
Modified Bland-Altman plots for MFBIA bias compared to DXA for arms, legs, trunk, and whole body fat mass (FM, upper panels) and fat-free mass (FFM, lower panels) assessments. men and women are represented by blue (circles) and red (triangles), respectively, dashed lines represent mean bias, dotted lines represent upper and lower limits of agreement.

Modified Bland-Altman comparisons are plotted in Fig. 3 and additionally described in Table 2 for bias ± SD of differences, and limits of agreement (LoA) between DXA and MFBIA measurements for both FM and FFM for each main body region (arms, legs, trunk, total). Mean bias between the methods for the total FM and FFM indicate that MFBIA systematically underestimated FM and overestimated FFM compared to DXA (men FM −3.72 ± 2.63 kg, LoA −8.88–1.44 kg, and FFM 3.38 ± 2.79 kg, LoA −2.09–8.86 kg; women FM −1.92 ± 1.83 kg, LoA −5.51–1.67 kg, and FFM 2.00 ± 2.17 kg, LoA −2.25–6.25 kg). The modified Bland-Altman (MFBIA bias to DXA; Fig. 3) shows an increasing positive bias with higher values of arm and leg FM; while in contrast, FFM showed increasing negative bias with higher arm and leg values. Both FM and FFM for the trunk do not have a clear skew positive or negative within values. Graphically, as expected due to propagation of errors, the total values for both FM and FFM have the largest range LoA, as they are the summed values from all regions.

Test-retest reliability assessments, collected over a week would reflect both biological and measurement method reliability. Both regional and whole body components measured by MFBIA had exceptional test–retest reliability (ICC ≥ 0.987) (Table 3). Table 3 also confirmed the DXA criterion measures to be highly stable over the week (ICC ≥ 0.990). Test-retest reliability was exceptionally high for fat measures by both DXA (ICC 0.990–0.998) and MFBIA (ICC 0.990–0.995). Of FM measurements, the lower reliabilities were seen for legs for both DXA (ICC = 0.990) and MFBIA (ICC = 0.990). Test-retest reliability of FFM measurements was exceptionally high for all compartments and regions by both the DXA (ICC 0.990–0.995) and MFBIA (ICC 0.987–0.994). Additionally, test-retest reliability of TBW as well for intra and extracellular water (ICW, ECW) for the MFBIA system was very high (ICC 0.993–0.996) (Table 3).

**Table 3:** Combined men and women assessment for test–retest reliability of dual-energy x-ray absorptiometry (DXA) and multi-frequency bioelectrical impedance analysis (MFBIA) measurements for repeated assessments for subset (*n* = 117).

	Region	Unit	DXA ICC	DXA Bias ± SD	MFBIA ICC	MFBIA Bias ± SD
Fat	Arms	kg	0.990	−0.01 ± 0.15	0.995	0.08 ± 0.43
	Legs	kg	0.990	−0.03 ± 0.47	0.990	0.13 ± 0.78
	Trunk	kg	0.997	−0.07 ± 0.48	0.993	0.17 ± 1.48
	Relative	%	0.996	−0.04 ± 0.95	0.993	0.24 ± 1.28
	Visceral	cm^2^	0.995	−1.04 ± 6.45	0.993	1.18 ± 6.23
	Total	kg	0.998	−0.04 ± 0.61	0.994	0.41 ± 2.41
Fat−Free	Arms	kg	0.992	0.04 ± 0.39	0.993	−0.10 ± 0.62
	Legs	kg	0.994	−0.12 ± 0.83	0.987	−0.16 ± 1.69
	Trunk	kg	0.990	−0.12 ± 0.93	0.994	−0.33 ± 1.68
	Total	kg	0.995	−0.16 ± 1.52	0.994	−0.59 ± 4.08
Water	Total	L	N/A	N/A	0.995	0.45 ± 2.59
	Intracellular	L	N/A	N/A	0.996	0.26 ± 1.57
	Extracellular	L	N/A	N/A	0.993	0.18 ± 1.12

**Note:** DXA dual-energy X-ray absorptiometry, ICC intraclass correlation coefficient (method reported for average of fixed raters, ICC(3k), MFBIA multi-frequency bioelectrical impedance analysis.

## Discussion

Compared to our previous laboratory-controlled study, the wider limits of agreement and higher MAPE values observed in this real-world setting suggest a greater degree of variability introduced by uncontrolled factors. These data validated the use of MFBIA as a practical and reliable method for estimation of whole body and regional FM and FFM composition. Fat was underestimated and FFM parameters were slightly overestimated, as previously reported by us and also reported for other MFBIA devices from other manufacturers (SECA, Tanita, Impedimed) [19, 23, 30–32]. It has been suggested that segmental analysis may explain, in part, this bias [16]. Several factors might contribute to this bias despite the high reliability. Previous reports have suggested that greater BMI may be associated with greater bias [30, 31, 33]. Several studies have attempted to develop correction factors for body sizes and shapes, and this may be helpful in smaller bodies (e.g., children) but has been less helpful for adults [34, 35]. We included an analysis of MAPE to assess variability of body composition components relative to the total mass or volume of tissue involved; limb fat and fat-free components were markedly higher than trunk and total body (Table 2) and the bias increased with mass (Figs. 1 and 3).

While the InBody 770 demonstrated exceptionally high test-retest reliability for FM, FFM, and TBW measurements (ICCs 0.987-0.996), indicating low technical error, it is important to distinguish reliability from accuracy. High reliability means the device provides consistent measurements under similar conditions. However, our data and the results reported by others [30, 31, 33], reveal a systematic bias compared to DXA, with FM being underestimated and FFM overestimated. This highlights that the device can be both highly reliable yet still exhibit consistent inaccuracies relative to a reference method. The bias suggests limitations within the underlying algorithms and assumptions of the MFBIA technology under varying biological parameters, rather than inconsistencies in the measurements themselves.

The InBody 770 VAT estimates were similar over the week, but values differed markedly from DXA VAT estimates. Both accuracy and precision were lacking in these comparisons to the criterion measure and in repeated measurements (Table 2, Fig. 2). The apparent basis for the BIA estimation is an electrical resistance that reflects both the truncal cross-sectional area (essentially an estimate of the circumference) and the contained visceral fat [36]. However, there remain some unexplained assumptions about the truncal subcutaneous fat layer [37]. The subcutaneous fat component varies with adiposity, sex, and age and these suggest potential predictive factors for the estimation of the subcutaneous layer, which could be subtracted from total truncal fat to obtain a VAT estimate. However, there is still a high variability among these factors [38]. Matsuzawa demonstrated this large variability in CT-determined VAT and subcutaneous fat between individual sumo wrestlers [39]. In the present data, higher total fat is associated with an increased variability in the BIA-determined VAT. One strategy to improve the BIA VAT assessment might involve the inclusion of some other geometry factors, such as a simple waist circumference [34, 35]. Defining VAT through electrical properties of the trunk tissues requires further research, and scientific explanation not currently provided with the proprietary algorithms. The use of DXA to estimate VAT has its own limitations because of technology and software issues, as highlighted by Kaul et al. [40] and Ashby-Thompson et al. [41]. Furthermore, BIA, whether SF or MF, lacks a clear physical definition for VAT measurement and has demonstrated poor agreement with MRI determinations [42]. These factors may explain the large MAPE values observed in our study and should result in cautious interpretation of VAT results. The particularly large MAPE values and discrepancies between InBody 770 and DXA VAT estimates highlight a significant limitation of this MFBIA technology. While the device demonstrates good reliability (via ICC) on repeated VAT measurements, it is limited in its ability to accurately estimate VAT compared to DXA (Table 2, Fig. 2). The BIA estimation relies on assumptions regarding truncal fat distribution and requires scientific explanation that is not currently available through the proprietary algorithms.

The early concerns of biological effects on BIA-derived body composition estimates were largely based on the sensitivity of SFBIA (50 kHz) methods to deviations from a consistent 73% hydration of the FFM [5, 43]. Additionally, as SFBIA is a simple model that seeks to interpret the human from a single cylinder, MFBIA stand-on systems mitigate this simplification by adding more dimensions (added cylinders) to better represent human geometry. The use of more than one frequency and the inclusion of reactance and phase angle with resistance measures could theoretically provide a more robust assessment of water compartments and cell mass less affected by deviations from assumed hydration [44, 45]. While our study showed reasonable agreement between TBW estimates from MFBIA and SFBIA in real-world conditions, it is important to note that both methods can differ from reference values obtained by tracer dilution. In studies with hemodialysis patients, Raimann et al. [13, 46] demonstrated systematic biases in both SFBIA and MFBIS (spectroscopy) compared to direct (deuterium dilution) and indirect (bromide/TBK) ‘gold-standard’ methods, while also showing the ‘gold standards’ differed between each other. Notably, they found SFBIA tended to overestimate TBW compared to MFBIS [46] and showed that MFBIS captured a significant treatment effect on extracellular fluid, while SFBIA did not [13]. These discrepancies may be explainable by several factors, including the influence of body composition and sex [13], with the underlying assumptions inherent in BIA algorithms (e.g., particular sensitivity to height), variations in tissue hydration, tracer binding properties, and model errors based on a design of a uniform cylindrical body [13]. Along with these complexities and lack of a true ‘gold standard’, comes the need for further research to refine BIA-based TBW estimations, to understand population-specific differences, and to revisit existing algorithms and models. Despite these errors, BIA is still clearly valuable for monitoring body fluid volumes and nutritional markers in clinical settings.

Data in this study compared reasonably well to data reported from a previous study with tightly controlled biological variables, including hydration status. The previous study involved a small sample of young fit individual soldiers under tightly controlled laboratory conditions [23]. Under these condition,s every BIA parameter had a better accuracy and test-retest reliability. Another test of this hypothesis was the comparison between the results of single and multi-frequency variability for the same individuals in this study. The MFBIA proprietary algorithm for TBW had a MAPE of 6% overestimation compared to the SFBIA TBW calculated from the 50 kHz resistance measurements (Table 2, Fig. 2). Unfortunately, these data reveal more about the comparison between algorithms and system engineering and less about the influence of hydration and other biological factors. Resistance was overestimated, *Xc* was underestimated, and *PhA* was closely aligned for summed segmental measurements obtained from the InBody system compared to the whole body values obtained from the SFBIA system (Fig. 2). Another value provided by the MFBIA (InBody) system for “total phase angle” significantly departed from the line of identity; no public information is available about which values are used in the body composition calculations (Fig. 2). Despite the potential for discrepancies in *PhA* values due to differences in conductor length and current pathways between MFBIA and SFBIA devices, we observed a relatively high correlation between the two methods. Segmental *PhA* values from the MFBIA are added together, which could result in values that are correlated but have an offset from the SFBIA values. This suggests that while the absolute *PhA* values may differ, the relative changes in *PhA* across individuals are consistent between the two methods. Additionally, segmental *PhA* may remain stable due to the localized nature of the measurement, which minimizes the impact of variations in conductor length and current pathways [16, 47].

From these data, we have to conclude again that standardization of test protocols is critical to the reliable application of BIA [48–50]. Some of the earlier methodological issues have been resolved through standardization of the human factors design of standing BIA devices that direct users to the correct positioning of arms and legs, removing variation in electrode placement [51]. We report here that everyday variation in prandial and hydration status as well as exercise had relatively little effect on the repeated measurements, but the measurements were not as accurate and reliable as a previous study from this laboratory using tight control of biological variables.

This study did not systematically challenge each of the biological variables of hydration, prandial status, recent exercise, and time of day but accepted the real-life variations represented in repeated measurements of a group of healthy fit young men and women going about their daily weekday routines on a military installation. Inquiries into specific biological factors that have the greatest effect on BIA assessments are needed. For example, Tinsley et al. showed an acute effect on BIA measurements for at least the first 10 min after bolus water consumption in standing subjects [9]. Another limitation to this study was the use of DXA in lieu of the gold standard but higher radiation and less practical CT scan. The use of DXA for body composition and regional assessments of the components of arms and legs, as well as estimation of VAT, does not represent the gold standard of CT assessment although it has previously been demonstrated to be a reasonable estimate of CT measurements [40]. DXA itself can be influenced at least by large variations in some of the same biological factors we considered, such as hydration status [52]. We acknowledge that we did not compare the body weight from the MFBIA device to the cumulative weight of components from DXA. Further research is needed to understand the potential impact of these differences on body composition estimates.

Our findings support the use of MFBIA systems for field epidemiological studies, military readiness standards, and clinical weight management. For these applications, assuming the use of one single device, reliability of measurement and reproducibility are more important than research-grade accuracy. Despite the underestimation of FM and over-estimation of FFM seen in this and other studies [19, 23, 30–32], previous studies have shown that BIA does track changes in body composition [53, 54], an important advantage over anthropometric methods which do not adequately track change [55, 56]. More than 30 years ago, researchers advocated for the replacement of cruder metrics of body composition, such as BMI, with BIA [57, 58]. Now there is adequate scientific support to replace BMI with BIA as a better assessment of actual body composition. In real-world conditions (i.e., relatively uncontrolled biological factors) MFBIA can provide a reliable and accurate method for assessing common regional and whole-body FM, FFM, and TBW. Assessments of limb composition are less accurate and reliable. VAT estimation did not match the DXA-estimated VAT and may not be any better than a simple waist circumference.

## Data Availability

Data and analyses from the current study are available from the corresponding author on reasonable request.
